# Molecular Evidence of Interhuman Transmission of *Pneumocystis* Pneumonia among Renal Transplant Recipients Hospitalized with HIV-Infected Patients

**DOI:** 10.3201/eid1010.040453

**Published:** 2004-10

**Authors:** Meja Rabodonirina, Philippe Vanhems, Sandrine Couray-Targe, René-Pierre Gillibert, Christell Ganne, Nathalie Nizard, Cyrille Colin, Jacques Fabry, Jean-Louis Touraine, Guy van Melle, Aimable Nahimana, Patrick Francioli, Philippe M. Hauser

**Affiliations:** * Hôpital de la Croix-Rousse, Lyon, France;; † Université Claude Bernard and INSERM U271, Lyon, France;; ‡ Hospices Civils de Lyon, Lyon, France;; § Hôpital Edouard-Herriot, Lyon, France;; ¶ Centre Hospitalier Universitaire Vaudois, Lausanne, Switzerland

**Keywords:** Epidemiology, Pneumocystis carinii, Pneumocystis jirovecii, interhuman transmission, cluster analysis, sulfa drug resistance, dihydropteroate synthase, single-strand conformation polymorphism, PCP, research

## Abstract

Ten *Pneumocystis jirovecii* pneumonia (PCP) cases were diagnosed in renal transplant recipients (RTRs) during a 3-year period. Nosocomial transmission from HIV-positive patients with PCP was suspected because these patients shared the same hospital building, were not isolated, and were receiving suboptimal anti-PCP prophylaxis or none. *P. jirovecii* organisms were typed with the multitarget polymerase chain reaction–single-strand conformation polymorphism method. Among the 45 patients with PCP hospitalized during the 3-year period, 8 RTRs and 6 HIV-infected patients may have encountered at least 1 patient with active PCP within the 3 months before the diagnosis of their own PCP episode. In six instances (five RTRs, one HIV-infected patient), the patients harbored the same *P. jirovecii* molecular type as that found in the encountered PCP patients. The data suggest that part of the PCP cases observed in this building, particularly those observed in RTRs, were related to nosocomial interhuman transmission.

## Abstract 2

Molecular evidence indicates that *P. jirovecii* may be nosocomially transmitted to severely immunosuppressed patients.

*Pneumocystis jirovecii* pneumonia (PCP) is a severe opportunistic infection in immunocompromised patients (*1**,**2*). It remains a major problem in some HIV-infected persons who are not receiving or not responding to highly active antiretroviral triple therapy and among those who are unaware of their HIV status. PCP is also of clinical importance in immunosuppressed patients, e.g., transplant recipients and those receiving chemotherapy for malignant diseases, who are not infected with HIV. Host specificity suggests that the reservoir of *P. jirovecii* is limited to humans. Primary infection in infants (*3**,**4*), as well as asymptomatic carriage by immunosuppressed persons (*5**–**8*), may serve as infectious reservoirs or sources in the community. Reactivation of a past infection was a postulate mechanism of infection in immunosuppressed patients, but de novo infection in recurrent episodes of the disease (*9*) has suggested that infection or reinfection from exogenous sources may occur. Horizontal airborne transmission has been demonstrated in several animal models (*10**–**12*).

Transmission of *P. jirovecii* from patients with active PCP to susceptible persons has been suspected in numerous descriptions of nosocomial clusters of PCP cases (*13**–**17*). Although a common environmental source of the infection was difficult to exclude, many patients in the clusters had contact with each other, which suggests that they may have transmitted *P. jirovecii* to one another. The early reports of PCP epidemics among malnourished children in orphanages and hospitals in the 1950s were also compatible with interhuman transmission of *P. jirovecii* (*18*). The strongest suspicion of transmission was provided by a case-control study performed for a cluster of five PCP cases in transplant recipients (*13*). This analysis showed that the patients had more encounters than matched controls at the outpatient facility with HIV patients who had or subsequently developed PCP. However, in these studies, transmission of *P. jirovecii* could not be assessed at the molecular level because no molecular typing method for *P. jirovecii* existed. Such methods were developed in the 1990s, and new clusters were analyzed. However, the few published anecdotal analyses often reported different genotypes within the clusters (*19**–**21*). Thus, interhuman transmission of *P. jirovecii* from PCP cases is still an open issue.

The latest guidelines developed by the U.S. Public Health Service and the Infectious Diseases Society of America for preventing opportunistic infections in persons infected with HIV state that although some authorities recommend that persons who are at risk for *P. jirovecii* pneumonia not share a hospital room with a patient who has PCP, data are insufficient to support this recommendation as standard practice (*22**,**23*).

In our molecular epidemiologic study, we investigated the possibility of *P. jirovecii* transmission between persons during a 3-year period in a hospital building that simultaneously hosted HIV patients (with and without PCP) and renal transplant recipients (RTR) (often during rejection episodes), and in which a cluster of PCP was observed.

## Material and Methods

### Hospital Setting

Edouard-Herriot Hospital is a 1,200-bed healthcare facility in Lyon, France, and is made up of several buildings. One of these buildings (building A, 80 beds) accommodates one hospital ward, an intensive care unit, an outpatient clinic, and a radiodiagnostic facility, which are mostly devoted to renal transplant medicine and clinical immunology, including HIV medicine. Another building (building B) hosts only patients with hematologic malignancies and is located 100 m away from building A.

### Data Collection

Our investigation included the 39 patients with PCP who were hospitalized in building A and whose bronchoalveolar lavage (BAL) specimen was available for molecular typing. These patients were chosen because interhuman transmission of *P. jirovecii* was suspected in this building. The database of the Department of Medical Information of the University Hospitals of Lyon was used to identify the demographic and clinical characteristics of the patients. Relevant data (prophylaxis regimen, hospitalization periods, dates of outpatient visits, immunosuppressive regimen) were also extracted from the medical charts of patients by using a questionnaire and log books of the outpatient clinic and radiodiagnostic facility.

### Laboratory Diagnosis and Storage of Specimens

PCP was diagnosed by using methenamine-silver nitrate (*24*) and Giemsa stains on BAL specimens in the parasitology laboratory of Claude-Bernard University, which has processed all specimens using the same techniques for many years. The number of BAL specimens submitted for patients seen at Edouard-Herriot Hospital has been stable over the years (1992–1998: 235, 241, 277, 254, 290, 215, 215, respectively). BAL specimens of patients with proven PCP were stored at –20°C.

### Molecular Typing

BAL specimens were typed as described previously (*25**–**27*) with the polymerase chain reaction (PCR)–single-strand conformation polymorphism (SSCP) method for typing *P. carinii*, now named *P. jirovecii* (*28*), in humans. The method consists of amplifying four variable regions of the *P. jirovecii* genome, followed by the detecting the polymorphisms with SSCP. The variable regions analyzed are the internal transcribed spacer 1 of the nuclear rDNA operon, the intron of the nuclear 26S rRNA gene, the variable region of the mitochondrial 26S rRNA gene, and the region surrounding the intron 6 of the β-tubulin gene. The different SSCP patterns observed are caused by one to four base-pair polymorphisms (*26*). A *P. jirovecii* type is defined by a combination of four alleles, which corresponds to the four genomic regions. If a specimen harbors two alleles of one or more of the four genomic regions, the patient was considered coinfected with two or more *P. jirovecii* types (*25*). For a given patient, each type is defined as an "isolate." Molecular typing was performed on specimens from patients in building A from 1994 to 1996, as well as on representative specimens collected during the same period in building B of the Edouard-Herriot hospital and in other university hospitals of Lyon. In addition, the dihydropteroate synthase (DHPS) genotype was determined by using PCR-SSCP as described (*29*). Four DHPS alleles have been described in *P. jirovecii* (*30*). The mutated alleles result in an amino acid change in the active site of the enzyme at position 55 (allele M1) or 57 (M2), or both polymorphisms (M3).

### Definitions

In the absence of knowledge of many biologic and epidemiologic characteristics of *P. jirovecii* infection, the incubation period of the not yet symptomatic patients and the period of infectivity of patients with PCP were postulated on the basis of available human and experimental data. Described clusters of PCP (*13**,**14*) suggest that the incubation period of de novo infection is 3–12 weeks. Accordingly, we assumed that a new infection with *P. jirovecii* (as opposed to reactivation) would occur 3–12 weeks before laboratory diagnosis of PCP. This finding is also in accordance with experiments in animals (*31**–**33*). Similarly, we considered that the risk of transmission from a *P. jirovecii*-infected patient to a susceptible one was likely to be highest from early symptoms to the middle of treatment. We assumed that an infected patient could transmit *P. jirovecii* from 3 weeks before to 2 weeks after the PCP diagnosis. We hypothesized that transmission was airborne and defined that a potentially infectious encounter occurred if a patient within his or her susceptible period and another patient within his or her infectious period visited the same location in building A on the same day. Transmission was considered possible if the patients encountered at least once and shared a common *P. jirovecii* type.

### PCP Prophylaxis, Isolation, and Immunosuppression

Four HIV-infected patients and four transplant recipients at risk for PCP were receiving sulfadoxine-pyrimethamine, but at a dosage lower than recommended for anti-*P. jirovecii* prophylaxis (25 mg pyrimethamine plus 500 mg sulfadoxine in one tablet taken once a week or every 2 weeks versus two tablets per week [*34*]). In addition, four HIV-infected patients were receiving aerosolized pentamidine (300 mg every 2 weeks). The other 27 patients did not receive any anti-*Pneumocystis* prophylaxis. A policy for isolating patients according to their underlying disease or to the occurrence of a PCP episode did not exist. Patients were allowed to move freely in the units and shared a TV room when permitted by their general condition. All RTRs, including those who experienced PCP, received usual inductive and maintenance treatments with prednisone, azathioprine, and cyclosporine. Treating rejection included high-dose corticosteroids and, if necessary, monoclonal antibodies.

### Case-Case Comparison

Case-case comparison based on molecular typing of the pathogen (*35*) was performed. Groups of cases infected with different *P. jirovecii* molecular types were compared to investigate differences in exposure histories.

## Results

**Figure 1:**
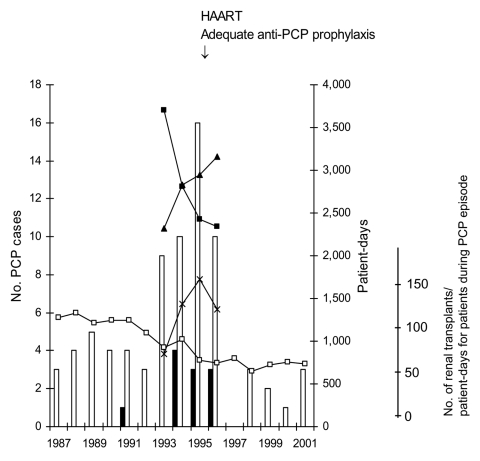
*Pneumocystis jirovecii* pneumonia (PCP) cases in HIV-infected patients (white bars) and in transplant recipients (black bars) at building A of Edouard-Herriot Hospital. Solid lines show the number of hospital patient-days for transplant recipients (filled squares), for HIV-infected patients (filled triangles), and for the patients during their PCP episode (crosses), as well as the number of renal transplantations performed (white squares). HAART, highly active antiretroviral therapy.

From 1994 to 1996, a total of 45 patients with 46 episodes of PCP were hospitalized in building A of the Edouard-Herriot Hospital. Their age ranged from 23 to 56 years (median 41), and most of them were male (82%). Thirty-six episodes were observed in 35 HIV-infected patients and 10 episodes in 10 RTRs. Thirty-one HIV-infected patients were admitted because of PCP, and PCP developed in all 10 RTRs and 4 HIV-infected patients during or shortly after hospitalization. These numbers represented a substantial increase compared to previous years, particularly in RTRs in whom only one case had been diagnosed during the 7 preceding years (Figure 1; the likelihood ratio for equality of two Poisson processes yields p = 0.00002; 1 case in 7 years versus 10 in 3 years). During the period from 1993 to 1996, the number of hospital patient-days for RTRs decreased by 37%, whereas those of the populations of HIV-infected patients and of patients with PCP increased, respectively, by 36% and 63% (Figure 1). The number of admissions of HIV-infected patients in building A was 339 in 1993, 364 in 1994, 401 in 1995, and 445 in 1996. The number of admissions of RTRs was 469 in 1993, 311 in 1994, 319 in 1995, and 297 in 1996. Precise admission figures before 1993 are not available, but the number of renal transplants performed at Edouard-Herriot Hospital has been decreasing from 128 in 1987 to 74 in 1996 and has been stable since then (Figure 1). The immunosuppressive regimen has not changed for RTRs from 1990 to 1997 in Edouard-Herriot Hospital.

### Molecular Typing *P. jirovecii*

**Figure 2:**
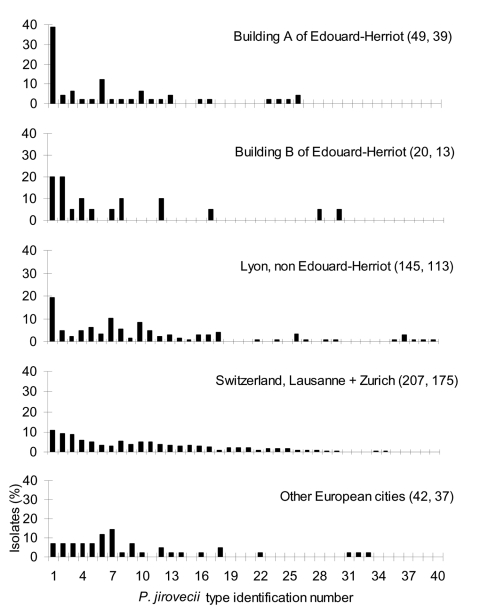
Frequency distribution of *Pneumocystis jirovecii* types observed in different cities and hospitals. Each type was considered as one isolate. The number of isolates followed by the number of specimens analyzed are indicated in the parenthesis for each geographic location. Data from Switzerland and other European cities are reproduced with permission from Hauser et al. 2001, AIDS 15(4):461–6 (*27*).

**Figure 3:**
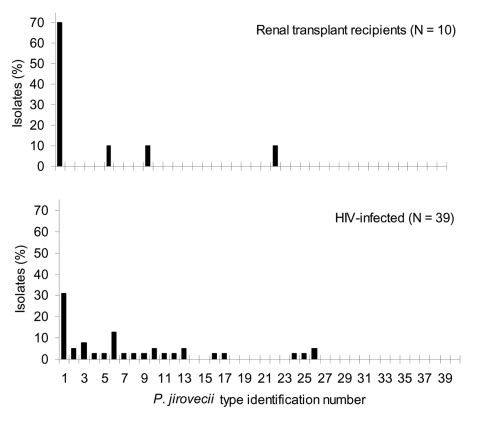
Frequency distribution of *Pneumocystis jirovecii* types observed in 30 HIV-infected patients and nine renal transplant recipients from 1994 through 1996 at building A of the Edouard-Herriot Hospital.

Thirty-nine of the 46 BAL specimens collected from 1994 to 1996 were available for typing (30 in HIV-infected patients and 9 in transplant recipients). Nineteen (49%) specimens corresponded to an infection with a single *P. jirovecii* type, 15 (38%) with two types, and 5 (13%) with more than two types. A total of 19 different *P. jirovecii* types were observed. In building A, the frequency of each type was 2%–12% of the *P. jirovecii* isolates, except for type 1 which represented 39% of the isolates and was isolated in 19 patients. Type 1, the most prevalent, represented 10%–20% of the isolates in Switzerland and other European cities (*27*), as well as in building B and other hospital facilities of Lyon (Figure 2). In particular, the frequency of type 1 was significantly higher in building A than in the other hospital facilities of Lyon (19 of 45 versus 28 of 145, Fisher exact test p = 0.003). Moreover, the frequency distribution of type 1 in the different categories of PCP patients hosted in building A was significantly different: it represented 31% (12 of 39) of the isolates from the HIV-infected patients, but 70% (7 of 10) of those from the transplant recipients (Figure 3, Fisher exact test p = 0.033). Seven of the 12 HIV-infected patients infected with *P. jirovecii* type 1 also harbored another type (coinfection), whereas one of the seven transplant recipients had a coinfection.

### Encounters and Exposures between Patients with *P. jirovecii*

From 1994 to 1996, 14 of 39 patients with PCP and available BAL specimens had prior encounters with patients with PCP (6 of the 30 HIV-infected patients and 8 of the 9 transplant recipients). A total of 118 potential encounters between patients with active PCP and patients who developed PCP 3–12 weeks after the encounter could be retrieved (Table). These 118 encounters corresponded to one or several encounters for the 14 patients. Among these 14 patients, PCP developed in 6 due to the same *P. jirovecii* type as 1 or 2 encountered PCP source patients, and *P. jirovecii* type 1 was involved in all 6 patients (5 transplant recipients, 1 HIV-infected patient). Of the 80 exposures involving *P. jirovecii* type 1, six PCP episodes were observed compared to no episodes of 38 exposures not involving *P. jirovecii* type 1.

**Table:** Cases with *Pneumocystis jirovecii* pneumonia (PCP) and potential encounters 3 weeks to 3 months before their PCP episode with other patients with active PCP

Date of PCP	Underlying disease^a^	*P. jirovecii* PCR-SSCP type	CD4 counts/mm^3^	No. of encounters with patients with active PCP	Total	With same *P. jirovecii* type	Presumptive nosocomial PCP^b^
1/10/94	RTR	1 and 6	–^c^	10	1	0	No
1/11/94	RTR	23	–	20	1	0	No
11/5/94	RTR	Undetermined	–	5	1	0	No
12/6/94	HIV	6	0	2	2	0	No
12/6/94	RTR	1	–	5	3	1	Yes
12/13/94	HIV	6 and 7	0	10	1	0	No
1/31/95	HIV	13 and 26	67	1	1	0	No
9/20/95	HIV	1	4	16	2	0	No
10/20/95	HIV	1	18	1	1	0	No
12/28/95	RTR	1	–	9	1	1	Yes
2/22/96	RTR	1	–	14	3	2	Yes
2/28/96	HIV	1	0	14	2	1	Yes
5/22/96	RTR	1	–	8	7	1	Yes
5/23/96	RTR	1	–	3	3	1	Yes
Total	8 RTRs, 6 HIV			118			5 RTR, 1 HIV

^a^PCR, polymerase chain reaction; RTR, renal transplant recipient; SSCP, single-strand conformation polymorphism. ^b^Nosocomial PCP was defined as PCP episode with exposure to one or several source patients infected with the same *P. jirovecii* type 3–12 weeks before the diagnosis. ^c^Data not available.

**Figure 4:**
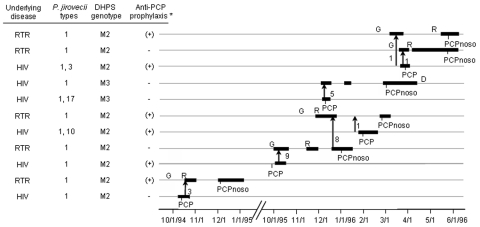
Potential encounters compatible with nosocomial interhuman transmission of *Pneumocystis jirovecii* at building A of the Edouard-Herriot Hospital (see Methods). Thicker parts of solid lines represent periods of hospitalization. Each encounter or consecutive encounters are figured by an arrow with the head indicating the direction of the presumed transmission, the number of encounters being indicated close to each arrow. *Anti-PCP prophylaxis was suboptimal. D, death. G, graft. R, rejection episode. RTR, renal transplant recipient. PCPnoso, nosocomial case.

Figure 4 shows the characteristics and chronologic events of the six putative nosocomial cases and their presumed source patients. It also shows the DHPS genotype. Five of the six nosocomial PCP patients harbored the M2 mutation and one the M3 mutation. Three nosocomial PCP patients harboring M2 mutation were receiving suboptimal anti-PCP prophylaxis. In all nosocomial cases, the presumed source patients had the same DHPS genotype. In some cases, an additional *P. jirovecii* genotype was recovered from the source patient, a finding compatible with coinfection with two types, one of which was not transmitted or present in a proportion sufficient to be detected in the nosocomial case. All the nosocomial PCP episodes in RTRs followed encounters which occurred when they were strongly immunosuppressed because of the treatment of a rejection episode (Figure 4).

Case-case comparison was used to compare exposure histories of two groups of patients, those harboring type 1 and those who did not. The proportion of patients who had at least one encounter during their susceptible period with a patient with active PCP harboring type 1 was higher in the first group (6/19 vs. 2/20), although not significant (p = 0.13, Fisher exact test). The frequency of the M2 mutation in patients not receiving any sulfa prophylaxis was significantly higher in building A than in other hospital facilities of Lyon (10/27 vs. 6/85, χ^2^ test p = 0.0004).

### Intervention

By mid-1996, all susceptible patients were placed on appropriate prophylaxis with co-trimoxazole (sulfamethoxazole plus trimethoprim), and HIV-infected patients had been started on highly active antiretroviral therapy. No PCP case was observed in 1997 (Figure 1), and no PCP cases were observed in transplant recipients as of December 2003.

## Discussion

During a 3-year period, 10 cases of PCP in transplant recipients occurred in a building of the Edouard-Herriot Hospital in Lyon, whereas only one case was observed in the preceding 7 years. These cases could not be attributed to improved diagnosis, change of immunosuppression regimen, an increase (a decrease actually occurred) of transplant recipients hospitalized in the facility. However, the outbreak occurred concomitantly with a progressive increase in the number of HIV-infected patients with and without PCP hospitalized in the same facility. Thorough molecular and epidemiologic analyses of the PCP cases showed the following facts: 1) transplant recipients, often in a stage of severe immunosuppression, shared the facility with HIV-infected patients with and without active PCP; 2) both transplant recipients and HIV-infected patients were receiving no or suboptimal anti-PCP prophylaxis; 3) *P. jirovecii* type 1 represented 70% of the isolates from the transplant PCP cases, but it represented <31% of the isolates in the HIV-infected patients with PCP in Lyon and elsewhere; and 4) all the transplant recipients (and some HIV patients) in whom PCP developed had been hospitalized in the facility at some point during the 3 months preceding their PCP episodes. Moreover, the proportion of patients exposed to *P. jirovecii* type 1 during their susceptible period was higher among cases infected with type 1 than among those not harboring type 1, although it did not reach statistical significance (p = 0.05). Finally, review of the medical charts indicated potential encounters between PCP cases during their susceptible period and other patients with active PCP (Table). Encounters with patients with a PCP episode involving the same *P. jirovecii* type, which may have led to transmission, were possible in 5 of the 10 transplant recipients and 1 of the 30 HIV patients. Taken together, these facts suggest that at least half of the PCP cases in transplant recipients (and possibly some in HIV patients) may be the result of a nosocomial acquisition of *P. jirovecii*.

However, alternative explanations exist that cannot be excluded. First, the transient presence of *P. jirovecii* in the air of hospital corridors has been described (*36*), which raises the possibility of an environmental source of *P. jirovecii* type 1 in building A. The following facts argue against this possibility: 1) the existence of a long-lasting environmental source of *P. jirovecii* has never been established, and 2) a high prevalence of type 1 was not observed in building B, which is located 100 m from building A and hosts patients with hematologic malignancies. Second, our study provides epidemiologic and molecular evidence that nosocomial transmission of *P. jirovecii* can occur, but whether this transmission would have occurred directly or indirectly through carriers is unclear. Indeed, carriage of *P. jirovecii* DNA has been described in the lungs of asymptomatic, immunosuppressed persons (*5**–**8*), as well as in the nose of immunocompetent relatives and healthcare workers in close contact with a PCP patient (*37*). Moreover, transmission by immunocompetent carriers to susceptible hosts has been demonstrated in the mouse model (*38*). Thus, indirect transmission through healthcare workers, physicians, or asymptomatic immunosuppressed patients cannot be ruled out.

For 14 of the 39 patients with PCP observed during the 3-year period, potential encounters with patients with active PCP during the 3 months preceding their episode had been documented (Table). However, only *P. jirovecii* type 1 was involved in encounters that apparently resulted in secondary cases. Part of this observation may be related to the higher prevalence of type 1 in Lyon (≈20%), although this could not explain the 40% rate of type 1 in building A. Another possibility is that type 1 might be more transmissible or virulent. This finding would be consistent with the fact that this type was one of the most prevalent types also in other geographic areas (Figure 2) (*27*). Moreover, specific *P. jirovecii* genotypes have been associated with more severe clinical symptoms (*39*) or with resistance to certain drugs (*40*). In our study, a mutation in the active site of DHPS was present in all six presumptive nosocomial PCP cases. The mutation may have favored acquisition of type 1 rather than another type by the three patients who were receiving suboptimal prophylaxis with Fansidar (Roche, Nutley, NJ). The presence of this mutation in the nosocomial PCP cases of our study suggests that *P. jirovecii* was acquired shortly before the episode because the frequency of DHPS mutations greatly increased only in the 1990s (*41*). Moreover, in patients not receiving any sulfa prophylaxis, the frequency of M2 mutation was significantly higher in building A than in other hospital facilities of Lyon (p = 0.0004), a fact that suggests nosocomial interhuman transmission of *P. jirovecii*.

Our study provides insight into the relative importance of nosocomial acquisition of *P. jirovecii* if infectious and susceptible patients are in close contact. Even though infected and susceptible patients were kept in unusually close proximity in this hospital, relatively few cases compatible with nosocomial interhuman transmission seem to have occurred. This finding suggests that transmission from patients with active PCP is limited, which is consistent with studies that we performed in HIV outpatient clinics that suggested infrequent cross-infections (*27**,**42*), as well as with a study comparing contact histories of patients with or without PCP (*43*). The source remains undetermined for the infection in the five transplant recipients for whom no potentially infectious encounters were found. One possibility is that carriers of *P. jirovecii* in the hospital have played a role.

The available data and the arbitrary definitions we had to use, in light of the absence of precise scientific data on *P. jirovecii* infection, are limitations of our study. We could not demonstrate that the presumed encounters actually occurred or define the precise nature of the encounter. Also, we could not firmly exclude other potential sources of *P. jirovecii*, such as the environment or asymptomatic carriers. Nevertheless, to our knowledge, this study is the first to suggest that *P. jirovecii* may be nosocomially transmitted and acquired by severely immunosuppressed patients. Given the increased number of reports relating resistance to anti-*Pneumocystis* drugs, prophylaxis of patients at risk might not be sufficient to achieve prevention. Moreover, prophylaxis is often not satisfactory because of secondary effects. Consequently, avoiding contact between persons at risk for PCP and patients with active PCP may be warranted and should be added to prevention guidelines.
